# The State of Microbiology Diagnostic of Prosthetic Joint Infection in Europe: An In-Depth Survey Among Clinical Microbiologists

**DOI:** 10.3389/fmicb.2022.906989

**Published:** 2022-06-20

**Authors:** Erlangga Yusuf, Charlotte Roschka, Jaime Esteban, Annibale Raglio, Anna Tisler, Philippe Willems, Tobias Siegfried Kramer

**Affiliations:** ^1^Department of Medical Microbiology and Infectious Diseases, Erasmus University Medical Center, Rotterdam, Netherlands; ^2^Institute of Hygiene and Environmental Medicine, Charité-Berlin University of Medicine, Berlin, Germany; ^3^Clinical Microbiology Department, IIS-Fundación Jiménez Díaz Foundation Health Research Institute, Universidad Autónoma de Madrid, Madrid, Spain; ^4^Unit of Microbiology and Virology, ASST Papa Giovanni XXIII, Bergamo, Italy; ^5^Institute of Family Medicine and Public Health, University of Tartu, Tartu, Estonia; ^6^Department of Microbiology, GZA Hospitals, Antwerp, Belgium; ^7^ADR Laboratory Group Dr. Kramer & Colleagues, Geesthacht, Germany

**Keywords:** prosthetic joint infection, clinical microbiology, culture, synovial fluid, sonication, reporting

## Abstract

**Background:**

This study aims to give an overview on how microbiology diagnosis tests of Prosthetic joint infections (PJI) is performed in Europe, and to explore whether any factor influences the decision on implementing a test.

**Methods:**

An extensive online survey of clinical microbiologists from seven European countries (Belgium, Estonia, Germany, Italy, Netherlands, Switzerland, and Spain). Following items were assessed: (i). general information on the laboratory, (ii) preference of the laboratory and clinical microbiologists regarding samples, (iii) transportation and (iv) processing of explanted foreign bodies and tissues and synovial fluid, (v) culture media and culture duration, (vi) reporting (identification and susceptibility testing), and (vii) use of molecular microbiology techniques.

**Results:**

Invited were 163 clinical microbiologists. The response rate from each country was above 50% (range 51–78%), except for Germany (36%). Frequent PJI diagnostics were the use of tissue pre-processing (58.1%), culturing synovial fluid in blood culture bottles (45.5%), use of sonication for processing explanted prosthesis (56.8%), reporting the presence of synovial leukocyte counts (67%), use of blood aerobic and anaerobic agar (97.7%), and enrichment media thioglycolate (69.3%). The most common incubation time of the culture media is 7–14 days (34.1–70.5%). The clinicians were called to report the culture results (80.7%), and to give antibiotic recommendation (67%).

**Conclusion:**

There are common practices in processing PJI samples and reporting results, which is promising for harmonization of PJI diagnostic in the future. However, variation in diagnostic tests should also be considered in interpreting and comparing clinical microbiology results.

## Introduction

Prosthetic joint infections (PJI) occur in 0.5–2% of the performed arthroplasties (Tande and Patel, 2014). Due to the aging population, the number of joint arthroplasty is predicted to be increased (Ackerman et al., 2019; Matsuoka et al., 2021). Consequently, it will increase the numbers of PJI. PJI is managed by a combination of antimicrobial therapy and surgery (Yusuf and Borens, 2015). The diagnostic procedure for PJI should be highly reliable because the invasive nature, and long duration of required treatment. To oversee the diagnosis, the role of clinical microbiology laboratories and clinical microbiologists is paramount.

There are several diagnostic microbiology approaches that are applicable mostly for PJI diagnosis and less in other types of infections, such as obtaining multiple tissue samples (Atkins et al., 1998) and releasing bacteria from the removed joint prosthesis by sonication (Trampuz et al., 2007), vortexing (Portillo et al., 2013), or addition of dithiothreitol (Sambri et al., 2018). Several novel but even less standardized laboratory tests in PJI diagnostic have also been introduced, such as microcalorimetry of synovial fluid (Morgenstern et al., 2020), and the application of minimal biofilm inhibitory concentration (MBIC) or minimum biofilm eradication concentration (MBEC) (Sandoe et al., 2006). Since their original publications, the diagnostic methods are continually evaluated, and the conclusion is not always unequivocal (Dudareva et al., 2018; Yan et al., 2018). Most clinical microbiology laboratories offer a selection of tests based on guidelines, recommendation, and own experience. The tests performed may thus vary.

Identifying the heterogeneity of diagnostic approaches for PJI is needed to understand variable results and consequently variation in the PJI prevalence. The lack of standardization of methods needs to be assessed too. The aim of this in-depth survey is to give an overview of on how microbiology diagnosis is performed in several European countries, and to explore which factor influences the decision on implementing a test.

## Materials and Methods

### Survey Strategy

The survey was conducted among clinical microbiologists in seven European countries (Belgium, Estonia, Germany, Italy, Netherlands, Switzerland, and Spain) in English language. Survey questions were developed by two clinical microbiologists (EY and TK). In September 2020, the survey was piloted among seven clinical microbiologists from the participating countries. The survey was validated by asking their co-workers to fill in the survey. The validation showed >95% concordance of the responses to the survey questions. These clinical microbiologists were further requested to prepare a list of potential clinical microbiologists participants for this survey. The potential participants should be representative of clinical microbiologists in their countries (i.e., working in various geographic areas and from academic and non-academic hospitals). The potential clinical microbiologist to be invited can only represent a single clinical microbiology laboratory. The invitation to participate was sent to the e-mails of possible participants using a personal link in June 2021. It was allowed to refer the invitation to a different colleague, technician or trainee in that laboratory. Perhaps due to the corona pandemic, the response rate in July 2021 was as low as 30% after three reminders within 3 weeks sent by e-mail. Therefore, it was decided to change the approach for non-responders, by sending them a personal email. The survey was closed 6 month after initial invitation.

### Survey Domains

This was an in-depth survey asking laboratory protocols in details. It was estimated that 30 min was needed to answer the questions. There were 32 questions divided into 7 sections (Supplementary File 1): general information regarding the laboratory and number of PJI diagnosis, preference of the laboratory and clinical microbiologists regarding the samples, transporting the samples (explanted foreign bodies and tissues), processing the samples (tissues, explanted foreign bodies, and synovial fluid), culture media and culture duration, reporting (identification and susceptibility testing), and molecular microbiology techniques.

### Statistical Analysis

Frequencies and percentages of variables were presented. Spearman and binary logistic regression analysis were performed to explore the correlation and association between the 2 variables, respectively. All analyses were performed using IBM SPSS Statistics for Windows, Version 26.0 (IBM Corp., Armonk, NY).

## Results

### Participant’s Characteristics

We invited 163 clinical microbiologists (18 from Belgium, 4 from Estonia, 39 from Germany, 26 from Italy, 30 from Netherlands, 11 from Switzerland, and 35 from Spain) to participate. The response rate from each country was above 50% (Belgium 78%, Estonia 75%, Italy 54%, Netherlands 60%, Switzerland 64%, and Spain 51%), except for Germany (36%). The responses from 88 clinical microbiologists from 88 laboratories were included (Table 1).

**TABLE 1:** Characteristics of clinical microbiologists and the laboratories participated in this survey.

Characteristics	*n* (%)
Countries	Belgium: 14 (15.9), Estonia: 3 (3.4), Germany: 14: (15.9), Italy: 14 (15.9), Netherlands: 18 (20.5), Switzerland: 7 (7.9), Spain:18 (20.5)
Accredited lab	77 (88)
Type of hospitals served	University hospitals only: 26 (29.5) Public hospitals only: 21 (23.9) Private hospitals only: 3 (3.4) Mixed university and public hospitals: 21 (23.9%) Other combinations: 17 (19.3)
Size of the largest hospitals served by the laboratory based on number of hospital beds	Less than 500: 17 (19.3) 501–750: 25 (28.4) 751–1,000: 25 (28.4) More than 1,000: 21 (23.9)
Presence of clinical microbiologists or infectious diseases specialists in the hospital	85 (96.6)
Distance from wards to clinical microbiology laboratory	In the same building: 34 (38.6) Within 10 km: 21 (23.9) Large (11–100 km): 32 (36.4)
Number of implanted prosthetic joint procedure per year	Less than 50: 7 (8.0) 51 to 100: 23 (26.1) 101 to 250: 29 (33.0) 251 to 500: 11 (12.5) More than 500: 12 (13.6)
Estimated number of prosthetic joint Infection specific samples per year	Less than 50: 12 (13.6) 51 to 100: 32 (36.4) 101 to 250: 22 (25.0) 251 to 500: 10 (11.4) More than 500: 5 (5.7)

### Samples and Transportation

Most (*n* = 71/88, 80.7%) of the laboratories had a protocol on sampling. The majority of clinical laboratories (*n* = 79/88, 89.8%) reported that the samples they received were mostly tissue biopsies (Table 2).

**TABLE 2:** Type of samples received in clinical microbiology laboratories.

	Never, *n* (%)	Sporadically, *n* (%)	In the majority of cases, *n* (%)
Tissues	1 (1.1)	8 (9.1)	71 (89.8)
Explanted joint implants	15 (17)	26 (29.5)	47 (53.4)
Synovial fluid	1 (1.1)	40 (45.5)	46 (53.4)
Synovial fluid in blood culture bottle	26 (29.6)	35 (39.8)	27 (20.7)
**Swabs (location sampled)**			
*Tissue*	27 (30.7)	33 (37.5)	28 (31.8)
*Prosthetic joint*	31 (35.2)	38 (43.2)	19 (21.6)
*Fistula*	18 (20.5)	49 (55.7)	21 (23.9)

Using logistic regression analysis, we found an association between the frequency of swabs of peri-operative tissue submitted to the laboratories with the distance between the hospital and the laboratory. Laboratory with the distance >10 km from the hospital had an odds ratio (OR) of 3.0 (95% CI 1.01–8.9) of frequently submitting swabs of operative tissue than laboratory situated in the same building as the hospital.

A total of 64 laboratories (72.7%) reported that they received tissue materials mostly in dry container, 34 (38.6%) mostly in saline solution, 9 in enrichment media (10.2%), and 3 (3.4%) in Ringer’s solution. Among the laboratories that received foreign body samples (*n* = 69, 78.4%), 55 reported that they received the material mostly in dry container, 20 mostly in saline solution, 5 in Ringer’s solution, 5 in broth, and 1 in Micro DTTtect (total count exceeds 100% because multiple answers were allowed).

### Processing Tissue Samples

Almost all labs (*n* = 86, 97.7%) had a protocol for processing tissue samples from patients with suspected PJI (Table 3). More than half (*n* = 50, 58.1%) of participating laboratories mentioned that they performed tissue pre-processing. To this end, the laboratories used various commercial instruments, such as Qiagen (Retsch) Tissue Lyser (*n* = 6), IKA Ultra-Turrax^®^ (*n* = 5), gentle MACS™ dissociator (*n* = 3), and Stomacher^®^ (*n* = 2).

**TABLE 3:** Protocols of the participating laboratories on processing the samples.

Type samples	Laboratories procedures	*n* (%)^a^
Tissues	Pre-processing	50 (58.1)
	Rolling the samples directly to agar	28 (32.6)
	Putting it into liquid media	7 (8.1)
	Vortexing and sonicating	1 (1.2)
Explanted foreign bodies	Sonication	50 (56.8)
	Dithiothreitol	5 (5.7)
Synovial fluid	Leukocyte counts automated cell counter	37 (42)
	Leukocyte counts microscopy	22 (25)
	Inoculating fluid into blood culture bottles	40 (45.5)

*^a^Total may not add to total number of participants (n = 88) since not all laboratories performed culture on the mentioned samples.*

The resulting solution from pre-processing of the tissues was then inoculated into agar and enrichment media (*n* = 39/50, 78%), to enrichment media only (*n* = 7, 14%), to agar media only (*n* = 3, 6%), and to blood culture bottle only (*n* = 1, 2%).

### Processing Explanted Foreign Bodies

The participating laboratories that processed explanted foreign body materials and performed sonication (*n* = 50/88, 56.8%), added Ringer’s or saline solution (*n* = 37/50, 74.0%). Most of these laboratories (*n* = 36/50, 72%) did not recommend pre-determined volume, but it was based on the size of the prosthetic and the container. Standardized recommended pre-determined volume used sonication procedure varied, up to 500 ml. The resulting fluid after sonication underwent centrifugation according to 24/50 (48%) of the laboratories processing these type of samples. Only a minority of the laboratories prescribed that the resulting sonication fluid should be inoculated to blood culture bottles (*n* = 17/50, 34%) and mostly they prescribed 10 ml (*n* = 10) to be inoculated in these bottles.

### Processing Synovial Fluid Samples

Among the laboratories that received synovial fluid and performed leukocyte counts in it (*n* = 59, 67%), 37 (62.7%) used automatic cell counter, while 22 (37.3%) used microscope. 29/40 (72.5%) laboratories that inoculated synovial fluid in blood culture bottle used no pre-determined volume, and 11 (27.5%) prescribed a pre-determined volume (mostly ≤ 5 ml).

### Culture Media, Duration, and Identification

Almost all participants laboratories used aerobic (*n* = 84/88, 95.5%) and anaerobic (*n* = 86/88, 97.7%) blood agar, and chocolate agar (*n* = 83/88, 94.3%) plates. Specific gram-negative agar was used by the half (*n* = 45/88, 51.1%) of the participating laboratories. Additional media used were chromogenic agar (*n* = 6/88, 6.8%), selective gram-positive agar (such as Columbia nalidixic agar, Columbia CAP agar, and mannitol salt agar) (*n* = 6/88, 6.8%), Sabouraud (*n* = 4/88, 4.5%). Only one lab mentioned using of selective CLED and yolk agar.

There is a wide variation on incubation duration of aerobic agar: 7–14 days (*n* = 33/88, 37.5%), 3–7 days (*n* = 33/88, 34.1%), and <3 days (*n* = 22/88, 25.0%). The variation was less for anaerobic culture incubation duration, where half of the participants cultured it for 7–14 days, and 33% (*n* = 29/88) for 3–7 days.

The most common enrichment media used were thioglycolate (*n* = 61/88, 69.3%), brain heart infusion (*n* = 30/88, 34.1%). Both 3 (3.4%) laboratories reported the use of fastidious and trypticase soy broth. Anaerobic supporting broth Schaedler and Wilkins-Chalgren were reported by 5 (5.7%) and 1 laboratory, respectively. The enrichment broth was incubated mostly for 7–14 days (*n* = 62/88, 70.5%). About 14 (15.9%) prescribed incubation duration 3–7 days (*n* = 15.9%) and only 5 (5.7%) prescribed duration > 14 days.

Blood culture bottles as enrichment media were used by 41/88 (46.6%) laboratories. They used aerobic (*n* = 33), anaerobic (*n* = 36), and pediatric bottle (*n* = 18). These blood culture bottles were incubated for 7–14 days (*n* = 30/59, 50.1%), 3–7 days (*n* = 25/59, 42.4%), only a small minority incubated it for more than 14 days (*n* = 4/59, 6.8%). We did not specifically asked for blood culture enrichment procedure for PJI diagnostic only, therefore the denominator (*n* = 59) was higher than the number of the laboratories using blood culture bottles as enrichment media (*n* = 41).

Almost all (*n* = 83/88, 94.3%) used MALDI-TOF and 20 (22.7%) laboratories mentioned that they also use biochemical tests for identification of microorganisms.

### Reporting (Identification and Susceptibility Testing), and Molecular Microbiology Techniques

The culture results were mostly reported semi-quantitatively (*n* = 54/88, 61.4%). The major part of the laboratories used automated antimicrobial susceptibility tests (*n* = 77/88, 87.5%), such as Vitek^®^ (*n* = 47), BD Phoenix™ (*n* = 15), and Microscan Walkaway (*n* = 15). 10 laboratories (11.3%) used disk diffusion only. EUCAST breakpoints were used by 79/88 (89.7%) of the laboratories. Of these 7 (8%) used the combination of EUCAST and CLSI breakpoints and 2 (2.3%) CLSI only. The antimicrobial susceptibility test (AST) was reported selectively by 53 (60.2%) of the laboratories. Only one laboratory reported the use of MBIC routinely, and two laboratories reported MBIC or MBEC in research setting.

The major part of the laboratories called the clinicians to report the culture results of presumed PJI (*n* = 71/88, 80.7%), and 59 (67%) also gave antibiotic recommendation. Around 3 quarter (*n* = 67/88, 76.1%) of the laboratories had an access to the clinical data of the patients, and 49 (55.7%) also participated in multidisciplinary meetings with clinicians. The larger the distance between the laboratory and hospital, the lower the odds ratio to have access to clinical data and the larger the size of the hospitals, the larger the odds ratio to have PJI multidisciplinary meetings (Table 4).

**TABLE 4:** Association between distance of the hospital and access to clinical data and between hospital size and multidisciplinary meetings.

		Odds ratio (95% confidence interval)	*p*-value
**Access to clinical data**			
Distance	Within the same building	1 (ref)	
	<10 km	0.2 (0.03–0.9)	0.03
	>10 km	0.1 (0.02–0.5)	0.01
**Multidisciplinary meetings**			
Number of beds	251–500	1 (ref)	
	501–750	4.8 (1.3–18.4)	0.02
	750–1,000	3.6 (1.0–13.4)	0.06
	>1,000	3.9 (1.0–15.3)	0.05

### Molecular Microbiology Methods

Less than half (*n* = 37/88, 42%) of the laboratories had the capacity to perform molecular microbiology tests. Most of them (*n* = 34/37, 91.8%) could perform 16s RNA, and 6 used commercial multiplex PCR tests (BioFire^®^, Unyvero, GeneXpert^®^). None of the participating laboratories used whole genome sequence in diagnosing PJI. The PCR test was never performed solely without cultures. Only two laboratories always performed the PCR test along with cultures. Most of the laboratories performed PCR upon request (*n* = 27/88, 30.7%) or when the culture was negative (*n* = 19/88, 21.6%).

## Discussion

There are several important findings from the survey. As expected, the microbiology procedures vary among the participating laboratories. However, there are several procedures that can be considered as typical clinical microbiology tests in diagnosing PJI, such as culture of the tissues and the prosthesis explants, where >50% of the participating laboratories processed these materials using tissue processing and sonication, respectively. Culture of synovial fluid and leukocyte count in it is also common. Mostparticipants incubated the materials for less than 14 days. Semi quantitative reporting was the most common method and the clinical microbiology results are phoned to the clinicians. Other common procedures are the use of MALDI-TOF for identification, automated antimicrobial susceptibility test, and the use of EUCAST breakpoints.

Clinical microbiology criteria from organizations, such as Musculoskeletal Infection Society (MSIS) (Parvizi et al., 2018), Infectious Diseases Society of America (IDSA) (Osmon et al., 2013), and European Bone and Joint Infection Society (EBJIS) (McNally et al., 2021), overlap (Table 5), but there are fine distinctions. MSIS and IDSA advised the requirement of at least 3 periprosthetic intraoperative tissue samples, but the maximum differs. MSIS asked the maximum of 5, while IDSA advised 5 or 6 without a maximum. Since tissues are obtained by invasive methods, efforts should be taken to maximize microbiological yield and to prevent contamination. The simplest procedure is by streaking and rolling tissues on solid culture media. However, this method does not expose the inside parts of the tissue to the surface of the media, and it may reduce the detection rate of the microorganisms. To increase the probability of detecting microorganisms, many laboratories performed pre-processing of the samples. The additional value of pre-processing tissues is still the matter of debate (Cai et al., 2021; Yusuf et al., 2021).

**TABLE 5:** Clinical microbiology criteria according to various organizations.

	MSIS (Parvizi et al., 2018)	IDSA (Osmon et al., 2013)	EBJIS (McNally et al., 2021)
**Culture requirements to be submitted**			
Intraoperative of tissues culture	Yes: at least 3 and no more than 5 periprosthetic specimen culture samples be taken and incubated in an aerobic and anaerobic environment	Yes: at least 3 and optimally 5 or 6 periprosthetic intraoperative tissue samples or the explanted prosthesis, aerobic and anaerobic culture	Not specifically mentioned
Preoperative synovial fluid	Not specifically mentioned but is mentioned in diagnosis criteria.	Yes: should be performed in all patients with suspected acute PJI unless the diagnosis is evident clinically and surgery is planned and antimicrobials can be safely withheld prior to surgery.	Not specifically mentioned but is mentioned in diagnosis criteria.
Requirement for explanted prosthesis to be submitted for culture	No	Yes: see above	Not specifically mentioned but in the criteria sonication was mentioned.
**Positivity culture definition**			
Intraoperative culture	(Not specifically mentioned whether the culture should be intraoperative only): >2 positive culture the same microorganism	>2 Positive culture the same microorganisms 1 ((virulent *Staphylococcus aureus*)	>2 Positive culture the same microorganism, or 1 (depending on definition levels)
Preoperative culture	The same identification as intraoperative culture	The same identification as intraoperative culture	The same identification as intraoperative culture
Explanted prosthesis	Not applicable		Quantitative >50 CFU/ml or >1 CFU/ml (depending on definition levels)
**Cell counts of synovial fluid positivity cut-off**			
Leukocytes (/mm3)	>3,000	No cut-off mentioned, synovial fluid cell count is not used as diagnostic criteria	>3,000 or >1,500 (depending on definition levels)
Polymorphonuclear cells (%)	>80		>80 or >65 (depending on definition levels)

Synovial fluid culture is mentioned as a criterion by MSIS, IDSA, and EBJIS, but only IDSA requested that synovial fluid should be performed in all suspected PJI cases. In our survey, only half of the participants received synovial fluid. Our survey question was not specified to ask about synovial fluid prior to the surgery. Arguably, the number of synovial fluid prior to surgery may be even lower. Synovial fluid in a period before surgery is the only microbiological sample possible at that time, but it is laborious to perform, is associated with contamination risk and possible complication, and has limited predictive value (Schulz et al., 2021). Moreover, the volume varies and sometimes it is insufficient for culture. These factors may cause reluctancy to perform synovial fluid culture. When no synovial fluid culture is submitted, one of the possible criteria diagnose PJI is missing. Consequently, PJI rate will be lower than when synovial fluid culture is available for culture.

Approximately half of the participants in this survey received explanted prosthesis on a regular basis. While IDSA and EBJIS mentioned culture of explanted prosthesis as criteria, MSIS does not. There is also subtle difference between the positivity criteria of explanted foreign body culture between these organizations. While IDSA uses a qualitative criterion, EBJIS uses quantitative. In practice, quantitative criteria are prone to variations. In the original paper on sonication in PJI, 50 colony-forming units (CFU) per plate were used as cut-off, as determined after ROC curve analysis (Trampuz et al., 2007). The protocol in this paper mentioned 400 ml Ringer’s solution to be added to the container contained removed prosthetic joint, and vortexing was performed prior to and after sonication. The incubation time of the media was up to 7 days. Our survey shows that most of the laboratories do not follow this protocol such the use of pre-determined volume to be added in the container. Quantitative reporting will also depend on whether centrifugation is performed.

Swabs are an inferior type of samples to make PJI diagnosis and should be discouraged. Fortunately, only 20–30% of the participants received swab samples in the majority of the cases. Swabs were more frequently used in settings with a larger distance between the hospital and the laboratory. Perhaps, clinicians consider swabs as a convenient sample when it needs to be transported for longer distances. An additional explanation could be a greater difficulty of communicational exchange.

The culture media were mostly incubated for 7–14 days in this survey. The prolonged incubation was recommended to detect slow growing bacteria, such as *Cutibacterium acnes* (Schäfer et al., 2008; Kheir et al., 2018). This duration is relevant for late PJI, but less for acute PJI. Yet, it is logistically impossible for a clinical laboratory to have separate protocol for acute and late PJI samples. A space constraint of blood culture incubation system due to prolonged incubation is also an issue. A possible intermediate approach is to prolong the incubation only if low grade infection possibility is communicated by the clinician to the laboratory. The authors are of the opinion not to extend the culture duration beyond 14 days due to possible contaminants.

Multidisciplinary meetings take places in the majority of the participant’s hospitals. Often, these meetings take place in larger hospitals perhaps due to logistic reason. Such meetings should also be encouraged in the smaller hospitals. Clearly, success of PJI diagnosis and treatment needs close collaboration between orthopedic surgeons, clinical microbiology specialists, and infectious disease specialists (Yusuf and Borens, 2015).

**FIGURE 1:**
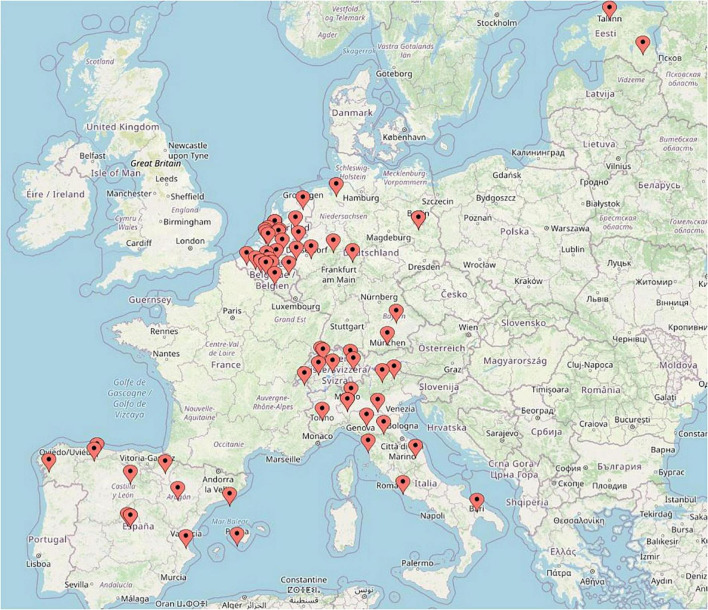
Geographic locations of participating laboratories in this survey.

The present study is the largest in-depth survey regarding PJI diagnostic, but there are several limitations. First, respondents were selected by among colleagues who worked regularly with PJI diagnostics, and this may somehow introduce bias. However, this selection was needed due to the ‘in depth’ nature of this survey. The participants are well balanced between academic and public hospital clinical microbiologists. The geography of participants seems also representative (Figure 1). Secondly, this survey did not assess new and innovative tests, such as microcalorimetry and biomarkers, in synovial fluid. MSIS uses biomarker criteria, such as alpha-defensin. Thirdly, the survey questions were not designed to differentiate samples from various surgical procedures (debridement, antibiotic and implant retention or single or multi-stage joint replacement), or to differentiate acute from late PJI. Yet, it is practically impossible for a clinical microbiology laboratory to make such a differentiation. It is also tempting to perform analysis on the differences between countries since health care system may influence the results. However, various size and number of participants per countries did not allow comparison to be made.

The findings from this survey can have several consequences of our findings. First, clinical microbiologists should be involved in the diagnostic process of PJI because reported culture results are results of complex processes which need some interpretation. Second, this study gives indication on ‘common’ diagnostic procedure for PJI in Europe. These common practices may help for harmonization of diagnostics approaches, in order to improve comparability and interpretation of reported results. Third, any variation in the PJI studies regarding prevalence and definition of PJI shouldbe interpreted in light of various laboratory methods. The PJI definition criteria used may be the same, but the laboratory procedures may be different.

In conclusion, this survey shows similarity and differences of clinical microbiology tests and consultation for PJI diagnostics and therapy in several European countries.

## Data Availability Statement

The raw data supporting the conclusions of this article will be made available by the authors, without undue reservation.

## Author Contributions

EY: conceptualization, methodology, formal analysis, investigation, resources, data curation, and writing—original draft. CR: conceptualization, methodology, resources, data curation, and writing—review and editing. JE, AR, AT, and PW: conceptualization, investigation, and writing—review and editing. TK: conceptualization, methodology, formal analysis, investigation, resources, data curation, and writing—review and editing. All authors contributed to the article and approved the submitted version.

## Conflict of Interest

The authors declare that the research was conducted in the absence of any commercial or financial relationships that could be construed as a potential conflict of interest.

## Publisher’s Note

All claims expressed in this article are solely those of the authors and do not necessarily represent those of their affiliated organizations, or those of the publisher, the editors and the reviewers. Any product that may be evaluated in this article, or claim that may be made by its manufacturer, is not guaranteed or endorsed by the publisher.
