# Supplementary material for: The State of Microbiology Diagnostic of Prosthetic Joint Infection in Europe: An In-Depth Survey Among Clinical Microbiologists
Source: Front Microbiol. 2022 Jun 20;13:906989. doi: 10.3389/fmicb.2022.906989 (PMC9251459; doi:10.3389/fmicb.2022.906989)
Supplement: Supplementary file 1 [file Data_Sheet_1.pdf]

ESGIAI questionnaire on microbiological diagnostic procedures for Prosthetic Joint Infection (PJI)

CHARITÉ UNIVERSITÄTSMEDIZIN BERLIN
Institut für Hygiene und Umweltmedizin

This tool has been developed to survey current practice in the diagnosis of PJI in microbiology laboratories.

Dear colleague,

Thank you very much for participating in this anonymous survey on diagnostic procedures in the laboratory for periprosthetic joint infection.
The objective of this project is to describe current practices among laboratories in different settings and extrapolate recommendations for a potential consensus.
The survey was initiated by ESCMID study group for implant infection (ESGIAI).
As a national delegate we kindly ask that you and one of your colleagues from your lab answer the questionnaire each independently.
This will improve validity of the project in the role out.

The length of the questionnaire was estimated with 30 minutes.
In case of questions please contact tobias.kramer@charite.de

Thank you for your work.

General Information: Your laboratory and the hospital(s) you serve

*** 1. In what country is your laboratory located? (single answer)**

- ◯ Belgium
- ◯ France
- ◯ Germany
- ◯ Italy
- ◯ The Netherlands
- ◯ Spain
- ◯ None of the above

*** 2. Please enter the first two letters of the town where your laboratory is located and the last two numbers of your zip code (e.g. Berlin 10551= BE51)**

[ ]

*** 3. What is your profession? (single answer)**

- ◯ Specialist in clinical microbiology
- ◯ Trainee in clinical microbiology
- ◯ Laboratory technician
- ◯ Other- please specify: [ ]

*** 4. Is your laboratory certified/accredited by an official organization? (single answer):**

- ◯ Yes
- ◯ No
- ◯ Don't know

* **5. What type of health care institution(s) does your laboratory serve? (select all that apply)**

- ☐ University/teaching hospital
- ☐ Public hospital
- ☐ Private hospital/clinic
- ☐ Nursing home
- ☐ General practitioner
- ☐ Other- please specify: ________

Your laboratory procedures regarding Prosthetic Joint Infection

* **6. Does your laboratory provide either a protocol or information to on sampling and other important factors for pre-analytics for cases of prosthetic joint infection? (single answer)**

- ◯ Yes
- ◯ No
- ◯ Don't know

* **7. What type of samples do you prefer to receive when diagnosing prosthetic joint infection? (multiple answers)**

- ☐ Synovial fluid
- ☐ Bone tissue
- ☐ Intraoperative swab of synovium
- ☐ Intraoperative swab of tissue (bone)
- ☐ Prosthetic joint or mobile parts
- ☐ Tissue other than bone
- ☐ Other- please specify: ________

* **8. When you receive tissue samples, approximately how often you also receive other types of samples from the same patient?**

	0–25% of cases	25–50% of cases	50–75% of cases	75–100% of cases	We do not receive this type of material
Synovial fluid	◯	◯	◯	◯	◯
Synovial fluid in blood culture bottle	◯	◯	◯	◯	◯
Tissue/bone biopsies	◯	◯	◯	◯	◯

	0–25% of cases	25–50% of cases	50–75% of cases	75–100% of cases	We do not receive this type of material
Prosthetic joint Explanted foreign bodies (Prosthesis, fracture fixation devices, e.g.)	○	○	○	○	○
Prosthetic joint swabs	○	○	○	○	○
Intraoperative tissue swabs	○	○	○	○	○
Fistula swabs	○	○	○	○	○

*** 9. How many tissue samples/biopsies do you receive on a regular basis in different containers/vials from a single patient? (single answer)**

- ○ We do not regularly receive tissue samples.
- ○ 1
- ○ 2–3
- ○ 4–6
- ○ more than 6

Transporting samples

*** 10. How are the explanted foreign bodies usually transported from the operating theatre to the laboratory? (multiple answers)**

- ☐ We do not regularly receive this type of sample.
- ☐ Dry in a sterile container
- ☐ In saline solution in a sterile container
- ☐ In Ringer’s solution in a sterile container
- ☐ In enrichment media (broth in a sterile container)
- ☐ In a solution of unknown type in a sterile container
- ☐ Other- please specify: ______

*** 11. How are tissue biopsies routinely transported from the operating theatre to your laboratory? (multiple answers)**

- ☐ We do not regularly receive this type of sample.
- ☐ Dry in a sterile container
- ☐ In saline solution in a sterile container
- ☐ In Ringer’s solution in a sterile container

- ☐ In enrichment media (broth in a sterile container)
- ☐ In a solution of unknown type in a sterile container
- ☐ Via a special tissue transport system (e.g. Port-A-Cul)
- ☐ Other- please specify: ______

Processing samples: Synovial fluid

*** 12. Does your lab count and differentiate the number of leukocytes in synovial fluid? (single answer)**

- ◯ Yes, qualitatively using a microscope
- ◯ Yes, automatically using an automatic cell counter
- ◯ No

*** 13. How much synovial fluid is plated on agar plates? (single answer)**

- ◯ No pre-determined amount, the number of drops varies
- ◯ We do not usually plate synovial fluid on plates.
- ◯ in µl: ______

*** 14. Do you inoculate synovial fluid into blood culture bottles? (single answer)**

- ◯ No, but we routinely receive synovial fluid in blood culture bottles.
- ◯ No, we do not usually inoculate or receive synovial in blood culture bottles.
- ◯ Yes. How much in ml? ______

Processing samples: Explanted foreign bodies

*** 15. Does your lab perform the following processes routinely on explanted foreign materials?**

	Yes	Uncertain	No
Dithiothreitol treatment (DTT)	◯	◯	◯
Sonication	◯	◯	◯

16. Which solution is added to the explanted foreign materials before sonication? (single answer)

- ◯ Saline solution
- ◯ Ringer’s solution
- ◯ Enrichment broth

- ◯ DTT
- ◯ Explanted foreign devices we receive are usually already in liquid which we cannot identify.
- ◯ We do not process this type of sample.
- ◯ Other- please specify: ____

*** 17. Do you centrifuge foreign body samples prior to plating in cases of suspected PJI? (single answer)**

- ◯ Yes, for DTT
- ◯ Yes, for sonicate fluid
- ◯ No
- ◯ We do not process this type of sample

18. Do you inoculate sonication fluid/DTT into blood culture bottles? (single answer)

- ◯ We do not process this type of sample.
- ◯ No
- ◯ Yes. How much in ml (per bottle)? ____

Processing samples: Tissue

*** 19. What process do you perform on tissue samples? (single answer)**

- ◯ Tissue is only rolled on the agar.
- ◯ Tissue is only rolled on the agar but is then put into another media (e.g. enrichment media, blood culture bottles).
- ◯ Tissue is pre-processed (e.g. by grinding). Sterile liquid (e.g. broth) is added and the agar is inoculated into this liquid.
- ◯ Tissue is pre-processed (e.g. by grinding). Sterile liquid (e.g. broth) is added and the agar or enrichment media is inoculated using the fluid.
- ◯ We do not regularly receive this type of sample.
- ◯ Other- please specify: ____

*** 20. Do you centrifuge tissue samples prior to plating in cases of suspected PJI? (single answer)**

◯ Yes ◯ No

*** 21. If you pre-process tissue samples, how much of the fluid obtained from processing is inoculated on the agar plate? (select one)**

- ◯ No pre-determined amount, the number of drops varies
- ◯ We do not pre-process tissue samples.

○ in ml: ______

Culture media and duration of culturing

*** 22. Which of the following agar media do you use for culturing in cases of suspected PJI? (select all that apply)**

- ☐ Blood agar plate
- ☐ Anaerobic blood agar plate
- ☐ Chocolate agar plate
- ☐ MacConkey
- ☐ Any chromogenic media
- ☐ Other- please specify: ______

*** 23. What type of broth do you use for culturing in cases of suspected PJI? (multiple answers)**

- ☐ We do not use enrichment media/ broth.
- ☐ Brain heart infusion
- ☐ Schaedler
- ☐ Thioglycollate
- ☐ Other- please specify: ______

*** 24. Do you use blood culture bottles as enrichment for cases of suspected PJI? (multiple answers)**

- ☐ We do not use blood culture bottles
- ☐ Pediatric blood culture bottle
- ☐ Anaerobic blood culture
- ☐ Aerobic blood culture

*** 25. Please choose the correct answers. (single answer)**

	less than 3 days	3–7 days	7–14 days	more than 14 days	We do not incubate those
How long do you incubate aerobic agar plates in cases of suspected PJI?	○	○	○	○	○
How long do you incubate anaerobic agar plates in cases of suspected PJI?	○	○	○	○	○

	less than 3 days	3–7 days	7–14 days	more than 14 days	We do not incubate those
How long do you incubate in blood culture bottles in cases of suspected PJI?	◯	◯	◯	◯	◯
How long do you incubate enrichment broth in cases of suspected PJI?	◯	◯	◯	◯	◯

*** 26. Do you routinely culture for difficult to culture organisms (Mycobacteria, Brucella,...) in cases of PJI? (multiple answers)**

- ☐ Yes, for every patient
- ☐ Yes, for patients with previous negative results
- ☐ No, only upon request
- ☐ No

Reporting, identification and susceptibility testing

*** 27. How do you identify bacteria? (multiple answers)**

- ☐ MALDI-TOF
- ☐ Biochemical tests
- ☐ Other- please specify: ______

*** 28. How do you report the identification? (multiple answers)**

- ☐ Qualitative (only the presence/absence of micro-organisms is reported)
- ☐ Semi-quantitative (categories, e.g. less than 50CFU/ml, more than 50CFU/ml)
- ☐ Quantitative (e.g. exact amount CFU/ml)

*** 29. How do you perform most Antimicrobial Susceptibility Testing for PJI? (single answer)**

- ◯ Disc diffusion
- ◯ Vitek
- ◯ MicroScan WalkAway
- ◯ Phoenix

- ◯ Merlin Micronaut
- ◯ Other- please specify:

* **30. What standard for A Antimicrobial Susceptibility Testing is used at your institution? (single answer)**

- ◯ EUCAST
- ◯ CLSI
- ◯ A combination of both (e.g. EUCAST filled in with CLSI)
- ◯ Other- please specify:

* **31. Are the following susceptibility tests performed in your lab?**

	Yes (routinely)	Yes, (as research)	No
Minimal biofilm-eradication concentration (MBEC)	◯	◯	◯
Minimal biofilm inhibitory concentration (MBIC)	◯	◯	◯

* **32. Which of the following activities are performed by the your lab with regards to prosthetic joint infection? (multiple answers)**

- ☐ Additional reporting by phone of results of a culture
- ☐ Treatment advice by phone regarding antibiotic recommendations
- ☐ Participation in multidisciplinary meetings on the treatment of prosthetic joint infection
- ☐ Selective reporting of results for Antimicrobials Susceptibility testing

Molecular diagnostic methods for periprosthetic joint infection

* **33. Are there any molecular microbiological methods available in your lab (e.g. PCR) that are routinely used for the diagnosis of prosthetic joint infection?**

◯ Yes ◯ No

Please use the following space to provide additional information or to comment on the laboratory diagnosis of prosthetic joint infection.

Submit
